# A Bishop's Ambition

**Published:** 1889-11-23

**Authors:** 


### The Hospital

A Weekly Journal of

Science, Medicine, Nursing, and Philanthropy.

*For Hospitals, Asylums, Medical Practitioners, Students, Nurses, Families, Parishes, Congregations, and the Charitable Public.*

Vol. VII. No. 165.] [November 23, 1889.

### A Bishop's Ambition.

The Bishop of Chester, speaking at a prize distribution at the Stockport Mechanics' Institution last week, said that one of his ambitions was "to write a first-rate novel." The Bishop has some understanding of the times in which he lives. The vast majority of the people of the present generation are willing to acquire new facts, and also to try to take newer and more reasonable views of things in general; they are, in short, quite willing to be instructed. But they are not willing to be bored. There is no particular reason why they should be bored if they can help it. What sort of a novel is it to be surmised the Bishop would like to write? "A first-rate novel," he himself says; and the first of all qualities in a first-rate novel is that it be readable, readable in the highest degree.

Now the Bishop of Chester has a comfortable stipend and a dignified position, and yet he is deeply anxious to become a novel writer. It is worth a moment's pause and thought to consider the peculiar longing of this particular episcopal mind. It is quite certain that he has no desire to compete for a place by the side of Dickens, or Thackeray, or George Eliot, or that most ecclesiastical of all novelists, the late Anthony Trollope. The fame of some of these, it is true, might well content even a bishop. But a bishop has his own line of things, and is usually best pleased if he can be successful therein. Why then does the Bishop of Chester, to repeat the question, long to write a first-rate novel? The Bishop has something in his mind which he desires to put clearly and intelligently into the minds of other people—something which he wishes them to understand fully; which he is anxious that they should thoroughly approve and deeply admire; which he would fain make their own mental property and a part of their daily lives.

It is to be presumed that the Bishop has materials out of which to construct his possible novel which have proved themselves deeply interesting to him, and which he feels assured would be deeply interesting to others if he could but give them life and actuality by means of persons and stage characters. Probably the only reason why the novel was not written long ago, is that he distrusted his own skill in constructing a story which should satisfy at once both his readers and himself. But it may be taken for granted that this occupant of the episcopal bench has a more urgent impulse to novel writing than the mere wish to interest his fellow-men. He undoubtedly feels that what he would like so much to put into the minds of possible readers is matter of deep and permanent importance and value. In a certain measure, the zeal of his convictions and desires is eating him up.

Well, then, here is a man, outside medicine and medical things, outside business and business things, outside Grub-street and Grub-street things, who is consumed with a desire to grapple with the minds of his reading fellow-countrymen and to make a change in those minds. The man is of sober temper, of adequate modern culture, of some scientific capacity. He is a phenomenon—a fact in nature and life. What is to be made of him and his ambition to write a novel, from a scientific point of view? Scientifically considered, a bishop's strong intellectual convictions are at least as intereresting as the shape of a bushwoman's head, or the question whether the equality in length of the middle and ring fingers denotes high mental development. How comes this bishop to hold and retain his belief in certain supposed facts and undoubted principles which are held by a few men of science to be not only incredible, but almost contemptible? The bishop is the equal in culture of the man of science, the equal in intellectual capacity, the equal in moral worth. Is it quite scientific to "pooh-pooh" him, and to hold him as a man not too sane in that he can believe things which in their own nature are said by a few to be not credible?

Some scientific men have climbed to a pedestal of all-round infallibility which they cannot continue to occupy. Science herself is the very outraged divinity who will, by-and-by, put forth her hand and pluck them down. There is science and science; the science of the surface, and the science of the depths; the science of the day, and the science of the aeons; the science of the flesh, and the science of the spirit; the science of the completed result, and the science of the energy, visible or invisible, which steadily and irresistibly works to its completion. Let the man of the scalpel, and the microscope, and the crucible tell the world what he sees and finds when he uses his tools and his methods with skill, and the world will be grateful to him. But when he presumes on the world's ignorance and gratitude to tell it what did or did not happen in ages of which he has had no experience, what can or cannot be done in regions of which he is scientifically as ignorant as the mollusc he dissects, then the world, recovering from its too great admiration of his former knowledge, calls him a guesser and an unscientific propounder of hypotheses which he never has subjected and never can subject to the test of scientific verification.

The great world has but tasted of the rare wine of science. A few have drunk more deeply, and their ill-balanced minds have become intoxicated with the unaccustomed draught. But as surely as the daybreak follows the darkness, so surely does the future of the world belong to science—to science and to such religions as are able to steady themselves on one and the same broadly scientific basis. Knowledge will advance; and as she advances she will pluck down and root out everything that cannot prove itself to be a necessary and vital part of the everlasting order. Crude and temporary scientific hypotheses will be thrown to the rubbish heap of exploded religious dogmas, and both of them will be burnt up and destroyed together.

What is the disposition of the medical mind towards the order that is coming? Is it narrowly scientific or broadly scientific? Or is it not scientific at all, but possessed and controlled by mere workshop intelligence? It is an undoubted fact that the average medical mind, with rare exceptions, is of the workshop order. What is more, many medical men pride themselves on the discreditable and disastrous certainty. Though bred in schools of science, they often learn nothing but the elements of a narrow and limited art. Not only are many of them utterly heedless of all that lies beyond the necessary knowledge of the hour, but even the broad vistas of light which the sciences they are compelled to study open out before them they do not follow, or care to enter upon.

Science is largely in the hands of medical men, and the world languishes and dies for want of its warm and vitalising light. But where is the medical man who is so consumed with an eager desire to impart his scientific knowledge to the world that he longs, with the zealous bishop, to write a novel for the purpose? Well may we ask, Where? What is the fact? That many medical men, even of the first eminence, regard with real or affected horror any attempt to enlighten the public mind on the elementary science of the physical man. The thing would be incredible if it were not true. What must the world think of such men of science? Either that they do not themselves believe in their own sciences; or that they are so shamelessly selfish as to wish to make them exclusively matters of merchandise; or that they are so stupid that they have not the necessary literary skill to make them "understanded of the people." Thus the world will judge; and it will do well. No class of men can isolate themselves and their work without injury both to their own characters and to the rest of the world. The days of exclusiveness, as of undiscovered continents, are past. The mind of the world insists upon possessing the whole world of knowledge, and that mind is a conqueror greater than Cæsar, before whom the strongest outworks of selfishness and exclusiveness must go down and be trampled underfoot. He who has eyes to see will adapt himself with all speed to the conditions of the new time. He who is wilfully blind and paralysed must of necessity be overwhelmed in the strife.

